# Optogenetic Control of the Mitochondrial Protein Import in Mammalian Cells

**DOI:** 10.3390/cells13191671

**Published:** 2024-10-09

**Authors:** Lukas F. J. Althoff, Markus M. Kramer, Benjamin Bührer, Denise Gaspar, Gerald Radziwill

**Affiliations:** 1 Faculty of Biology and Signalling Research Centres BIOSS and CIBSS, University of Freiburg, 79104 Freiburg, Germany; lukas.althoff@biologie.uni-freiburg.de (L.F.J.A.); markus.kramer@cibss.uni-freiburg.de (M.M.K.); benjamin.buehrer@uniklinik-freiburg.de (B.B.); denise.gaspar@bioss.uni-freiburg.de (D.G.); 2 Spemann Graduate School of Biology and Medicine (SGBM), University of Freiburg, 79104 Freiburg, Germany

**Keywords:** optogenetics, mitochondrial import, matrix peptidases, CRY2, LOV domain, MTS, TEV

## Abstract

Mitochondria provide cells with energy and regulate the cellular metabolism. Almost all mitochondrial proteins are nuclear-encoded, translated on ribosomes in the cytoplasm, and subsequently transferred to the different subcellular compartments of mitochondria. Here, we developed OptoMitoImport, an optogenetic tool to control the import of proteins into the mitochondrial matrix via the presequence pathway on demand. OptoMitoImport is based on a two-step process: first, light-induced cleavage by a TEV protease cuts off a plasma membrane-anchored fusion construct in close proximity to a mitochondrial targeting sequence; second, the mitochondrial targeting sequence preceding the protein of interest recruits to the outer mitochondrial membrane and imports the protein fused to it into mitochondria. Upon reaching the mitochondrial matrix, the matrix processing peptidase cuts off the mitochondrial targeting sequence and releases the protein of interest. OptoMitoImport is available as a two-plasmid system as well as a P2A peptide or IRES sequence-based bicistronic system. Fluorescence studies demonstrate the release of the plasma membrane-anchored protein of interest through light-induced TEV protease cleavage and its localization to mitochondria. Cell fractionation experiments confirm the presence of the peptidase-cleaved protein of interest in the mitochondrial fraction. The processed product is protected from proteinase K treatment. Depletion of the membrane potential across the inner mitochondria membrane prevents the mitochondrial protein import, indicating an import of the protein of interest by the presequence pathway. These data demonstrate the functionality of OptoMitoImport as a generic system with which to control the post-translational mitochondrial import of proteins via the presequence pathway.

## 1. Introduction

Mitochondria, known as the power houses of eukaryotic cells, generate energy in the form of ATP. Apart from their role in modulating the energy metabolism, mitochondria regulate the biosynthesis of amino acids, lipids, and cofactors, produce reactive oxygen species, control apoptosis, and integrate the cellular metabolism in general [1]. The human mitochondrial proteome comprises about 1300 proteins [2,3,4]. However, the mitochondrial genome encodes for only 13 proteins, all subunits of respiratory chain complexes [5]. All other mitochondrial proteins are nuclear-encoded, translated on ribosomes in the cytoplasm, and subsequently translocated to mitochondria. Five main pathway mechanisms distribute these proteins to the sub-mitochondrial place of their function: the outer mitochondrial membrane, the intermembrane space, the inner mitochondrial membrane, and the mitochondrial matrix [6,7,8]. The main pathway, covering 60–70% of mitochondrial protein import, is the presequence pathway depending on an N-terminal mitochondrial targeting sequence (MTS). An MTS consists of about 20–60 amino acid residues forming an α-helical structure with positively charged residues on one side of the surface and hydrophobic residues on the opposite side and recruits the linked protein sequence to the translocase of the outer membrane (TOM) complex [9,10,11]. After binding to the receptor subunits of the TOM complex, the MTS containing proteins are transported through the pore-forming subunit Tom 40 and transferred to the translocase of the inner mitochondrial membrane (TIM) complex [12]. The proteins are pulled through the cavity of the TIM 23 complex [13] with the help of a presequence translocase-associated motor (PAM), consisting of the mitochondrial chaperone mtHSP70 and co-chaperones [14,15]. This process is coupled to the membrane potential across the inner mitochondrial membrane, generating the proton motoric force (PMF) [16].

The mitochondrial processing peptidase (MPP) cleaves the N-terminal MTS, and other matrix peptidases can further process these proteins [17,18]. The presequence pathway is responsible for almost all matrix proteins and the proteins localized to the inner mitochondrial membrane.

Ribosomes connected to the outer mitochondrial membrane enable the co-translational import of proteins and regulate the dual targeting of proteins that function in mitochondria and other subcellular compartments [19,20]. However, the majority of mitochondrial proteins are synthesized on free cytosolic ribosomes and post-translationally recruited to the TOM complex supported by cytoplasmic chaperones HSP70 and HSP90 [21].

**Figure 1:**
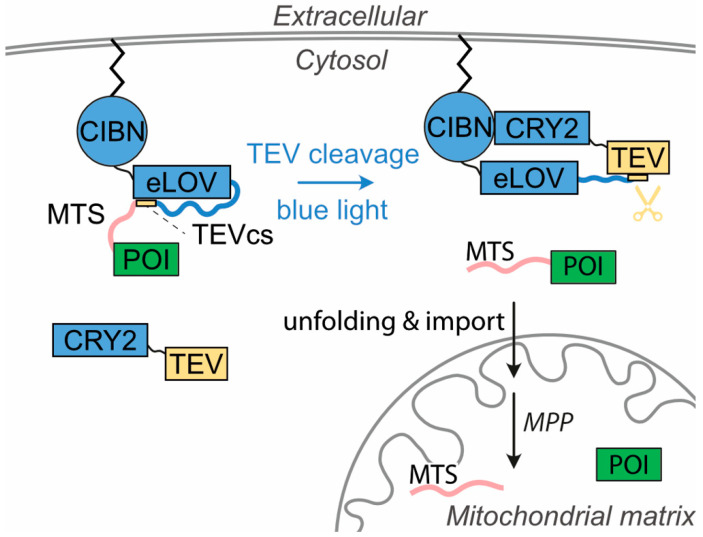
Principle of the optogenetic system OptoMitoImport. The system is based on the co-expression of the cytosolic photoreceptor CRY2 fused to the TEV protease and a membrane-anchored (m/p) [26] construct containing the CRY binding protein CIBN, the light switch eLOV, N-terminally linked to the TEV cleavage site (TEVcs), and a mitochondrial targeting sequence (MTS), followed by the protein of interest (POI). Upon blue light exposure, (1) CRY2-TEV recruits to the membrane-anchored fusion construct by interacting with CIBN; (2) the TEVcs is uncaged from eLOV; (3) TEV cleaves at the TEVcs and releases MTS-POI; and (4) MTS recruits to the TOM complex and enables the mitochondrial import of the POI. Matrix processing protease (MPP) cuts off the MTS, and the POI can exert its function in the mitochondrial matrix.

Optogenetics is an emerging field in synthetic biology [22,23,24]. The number of optogenetic tools engineered to control biological functions is steadily increasing [25,26,27,28]. In addition, the first therapeutical applications have been described [29,30]. Here, we developed a generic optogenetic tool, OptoMitoImport, to study and to control the post-translational import of mitochondrial proteins via the presequence pathway (Figure 1). This system is based on the work of Sanchez and Ting, which was established to improve the catalytic efficiency of the tobacco etch virus protease (TEV) and to control the expression of transcriptional reporters using two optogenetic switches controlled by blue light [31].

The switch formed by cryptochrome 2 (CRY2)/CRY2-interacting binder N-terminus (CIBN) [32] is used to recruit the TEV to its target protein carrying a TEV cleavage site (TEVcs). The improved light–oxygen–voltage-sensing domain (eLOV) cages TEVcs in the dark and releases it upon illumination. OptoMitoImport comprises two components: first, a cytosolic TEV fused to CRY2; and second, a plasma-membrane-anchored fusion construct carrying CIBN, an eLOV, C-terminally linked to TEVcs, and an MTS followed by the protein of interest (POI), which should be imported into mitochondria. Upon blue light exposure, (1) CRY2-TEV is recruited to the membrane-anchored fusion construct by interacting with CIBN; (2) the eLOV uncages TEVcs; (3) the TEV cleaves at TEVcs and releases MTS-POI; and (4) MTS recruits to the TOM complex and enables the mitochondrial import of the POI.

## 2. Materials and Methods

### 2.1. Cloning

DNA was amplified by PCR with Q5 DNA polymerase (NEB) according to the manufacturer’s protocol using 25 to 35 cycles. Primers were designed for Gibson cloning with an PCR annealing temperature of 65 °C. PCR products were separated on a 1% agarose gel and purified with a PCR extraction kit (Qiagen, Venlo, The Netherlands) according to manufacturer’s protocol. All constructs were cloned via Gibson cloning into the vector pcDNA3.1^+^ (Thermo Fisher Scientific Inc., Waltham, MA, USA). An aliquot of the Gibson assembly mix was used to transformed *E. coli* Top 10 (Thermo Fisher Scientific Inc., Waltham, MA, USA). Single clones were picked and expanded for plasmid extraction (Promega, Fitchburg, WI, USA) according to manufacturer’s protocol and followed by sequencing (Eurofins Scientific SE, Luxemburg, Luxembourg).

### 2.2. Cell Culture and Transfection

HEK 293T cells (DSMZ, catalog no. ACC 635) and HeLa cells (DSMZ, catalog no. ACC 57) were cultivated in DMEM + GlutaMAX^TM^ + 4.5 g/L D-glucose (Thermo Fisher Scientific) supplemented with 10% FCS (PAN), 100 Units/mL penicillin, 100 µg/mL streptomycin (Thermo Fisher Scientific) at 37 °C, and 5% CO_2_. DNA was introduced via transfection with polyethylenimine (PEI; Polysciences Inc, Warrington, PA, USA), as described before [27], using a DNA–PEI ratio of 3.3:1 with a total DNA amount of 5 µg (10 cm plate), 1 µg (6-well format), or 0.5 µg (24-well format). Cells were seeded with a density of 0.5 × 10^5^ cells cm^−2^ 20–24 h prior to transfection. Plasmid DNA, diluted in Opti-MEM (Thermo Fisher Scientific), was incubated with PEI for 15 min before the transfection mix was added dropwise to the cells.

### 2.3. Illumination

Twenty-four hours after transfection, cells were illuminated with either 450 nm or 465 nm depending on the illumination device and plate format. Experiments, including more than two illumination conditions not succeeded by fractionation or microscopy, were performed in the 24-well format using the optoPlate-96 system with the configuration software optoConfig-96 (Version 1.0.5) [33,34], where cells were irradiated with 465 nm at 2 or 10 W/m^2^ between 15 min and 10 h. All other experiments were conducted in 10 cm dishes, 6-well plates or 24-well plates, where irradiation was performed in the opto-Box format [27] with 450 nm light at 2 or 10 W/m^2^ for 4 or 7 h, depending on the experiments. Cells were incubated under dark conditions between transfection and the start of illumination. Illumination was performed such that all illumination conditions were terminated at the same time so that lysis could be performed in parallel for all samples in one experiment.

### 2.4. Cell Lysis

Cell lysis was performed 24 h after transfection or after the irradiation of cells with ice-cold lysis buffer (100 mM NaCl, 1 mM EDTA, 0.5% Triton X-100, 0.1% SDS, 20 mM Tris-HCl, pH 7.5, 0.2 µL/mL Benzonase). Lysate was incubated at −80 °C for 10 min, thawed on ice, and scraped of plates. The lysate was centrifuged for 12 min at 10,000× *g* at 4 °C. The samples were adjusted to 1× sample buffer (10% Glycerol, 62.5 mM Tris-HCl (pH6.8), 0.01% bromophenol blue, 2% SDS, 2.5% 2-mercapto ethanol) for western blot analysis. In the case of the experiment containing optogenetic components, cell lysis was performed in a red-light environment.

### 2.5. Mitochondrial Fractionation and Proteinase K Digestion

Fractionation was performed with the Mitochondria Isolation Kit for cultured cells (Thermo Fisher Scientific) according to the manufacturer’s protocol with the following changes: after centrifugation with 700× *g* for 10 min, the supernatant was transferred to a new reaction tube and centrifuged with 1200× *g* for 10 min; again, the supernatant was transferred to a new reaction tube and the manufacturer’s protocol was continued by centrifuging with 12,000× *g* for 15 min; finally, the mitochondrial pellet was lysed in lysis buffer.

The experiments including proteinase K treatment were performed as described above except that the buffers did not include protease inhibitors. Mitochondrial pellets were resuspended in 150 µL cold KPBS (136 mM KCl, 10 mM KH_2_PO_4_, pH 7.25) and separated into three samples for treatment with 20 µg/mL proteinase K with and without 0.5% Triton X-100 and a negative control without treatment before incubating at RT for 15 min. All samples were treated with 2.5 mM PMSF.

### 2.6. Depletion of the Membrane Potential

Cells were seeded and transfected as described above. At the start of illumination (dark sample one hour before lysis), growth media were changed for media containing a mixture of 0.8 µM antimycin A, 0.05 µM valinomycin, and 2 µM oligomycin (AVO). Cell lysis was performed as described above.

### 2.7. SDS PAGE and Western Blotting

SDS PAGE was performed in the BioRad Mini-PROTEAN^®^ format with 12% SDS separating gel and 5% SDS stacking gel with 90–120 V constant voltage. The proteins were blotted onto a PVDF membrane (Merck KGaA, Darmstadt, Germany) in a semi-dry format using a modified Towbin buffer with 20% EtOH prior to blocking in 5% dry milk in TBS-T for 1 h. After washing with TBS-T, the membrane was incubated with the respective primary antibody: anti-GAPDH (Cell Signal Technology, Danvers, MA, USA, #5174s, 1:5000 in 1% dry milk in TBS-T), anti-SDHA (Cell Signal Technology, #11998s, 1:5000 in 1% dry milk in TBS-T), anti-mtATP6 (Cell Signal Technology, #70262s, 1:1000 in 1% BSA in TBS-T), and anti-PDHK1 (Abcam, Cambridge, UK), #ab110025, 1:5000 in 1% dry Milk in TBS-T), incubating overnight at 4 °C. The secondary or HRP-linked primary incubation used were as follows: anti-HA-HRP (Cell Signal Technology, #2999s, 1:3000 in 1% dry milk in TBS-T), anti-Rabbit-HRP (Cell Signal Technology, #7074s, 1:3000 in 1% dry milk in TBS-T), anti-Mouse-HRP (Cell Signal Technology, #7076s, 1:3000 in 1% dry milk in TBS-T), incubating for 1 h at RT. Imaging was performed with ImageQuant LS4000 mini (Fuji, Minato, Tokyo, Japan) using SuperSignal^TM^ West Pico (Thermo Fisher Scientific) as an ECL substrate.

### 2.8. Fluorescence Microscopy

HeLa cells were seeded with 0.25 × 10^5^ cells cm^−2^ on untreated glass cover slips in a 24-well format and transfected with 0.5 μg DNA using Lipofectamin^TM^ 3000 transfection reagent (Thermo Fisher Scientific) according to the manufacturer’s protocol. HEK 293T cells were seeded on Ibidi µ-Slide 8 Well Grid-500 (cat nr. 80826-G500) with 0.6 × 10^5^ cells cm^−2^ and transfected with a total of 0.3 µg DNA per well using PEI as a transfection agent, as described before. Illumination was performed as described above 36 h after transfection in the opto-Box format with 2 W/m^2^ for 1 or 7 h. For the staining of the mitochondrial network (100 nm MitoTracker^TM^ Red CMXRos, Thermo Fisher Scientific) and the conjugation of Snap-tag with fluorescent dye (2.5 µM SNAP-Cell^®^ Oregon Green^®^, New England Biolabs, Ipswitch, MA, USA), the media were changed 30 min prior to the fixation of cells in 4% paraformaldehyde with subsequent incubation for 15 min at RT. Permeabilization was performed via the incubation of cells in permeabilization buffer (PBS with 2% BSA and 0.1% Triton X-100) for 30 min (HEK 293T) or 1 h (HeLa) at RT. HEK293T cells were subsequently imaged. In HeLa cells, nuclear staining was performed by incubating cover slips in 500 µL staining solution (4′,6-Diamidin-2-phenylindol 1:5000 in PBS) for 15 min at RT. The cover slips were washed twice in PBS and once in dH_2_O prior to mounting on slides with 7 µL mowiol (Carl Roth GmbH & Co. KG, Karlsruhe, Germany). Image acquisition was carried out with Zeiss LSM 800 Examiner/Fast Airyscan, Carl Zeiss AG, Oberkochen, germany upright microscope at excitation wavelengths 405 nm (DAPI), 488 nm (SNAP-Cell^®^ Oregon Green^®^), and 561 nm (MitoTracker^TM^). Emission was observed at 444 nm (DAPI), 521 nm (SNAP-Cell^®^ Oregon Green^®^), and 623 nm (MitoTracker^TM^).

## 3. Results

### 3.1. Characterization of Mitochondrial Signal Sequences

**Figure 2:**
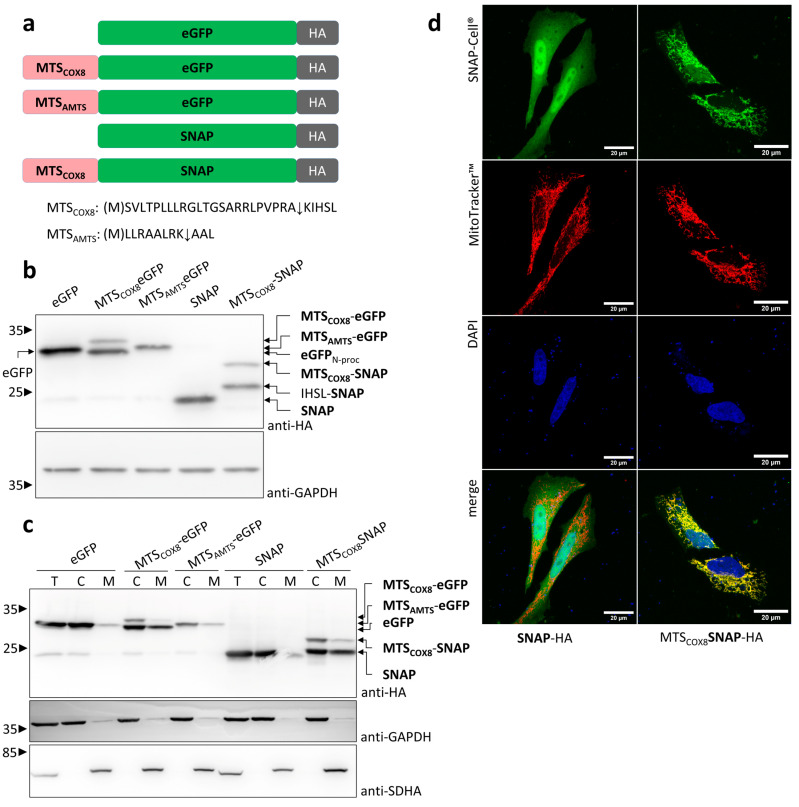
Functional analysis of mitochondrial targeting sequences: (**a**) Scheme of the MTS constructs. eGFP and SNAP served as reporter proteins linked to an MTS. MTS_COX8_ represents the N-terminal 28 residues of COX8 [35]. MTS_AMTS_ represents an artificial MTS of 11 residues [36]. Methionine, as the first residue during translation, is shown in parenthesis. All constructs carry a C-terminal HA-tag. The putative cleavage site for the mitochondrial processing peptidase MPP is marked by an arrow head. (**b**) HEK 293T cells were transfected with the constructs as indicated. Twenty-four hours after transfection, cells were lysed, and the lysates were subjected to SDS-PAGE followed by immunoblotting with anti-HA antibody and with anti-GAPDH as a loading control. (**c**) HEK 293T cells were transfected with the constructs as indicated and lysed 24 h after transfection. Proceeding from total cell lysates (T), cytosolic (C), and mitochondrial (M) fractions were prepared. Samples were subjected to SDS-PAGE and immunoblotted with anti-HA antibody, as well as with anti-GAPDH antibody as marker for the cytosolic fraction and anti-SDHA antibody as marker for the mitochondrial fraction. Each point shown in the experiments is repeated at least three times, albeit in different sets of an experiment. (**d**) HeLa cells transfected with constructs expressing SNAP-HA or MTS_COX8_-SNAP-HA were cultivated for 24 h. Before fixation with paraformaldehyde and analysis via confocal fluorescence microscopy, the cells were incubated with SNAP-Cell^®^ Oregon Green dye and MitoTracker TM Red CMXRos dye for the visualization of SNAP in green and the mitochondrial network in red in living cells, respectively. Co-localization is visualized by merging the images. DAPI was used for nuclear staining. Scale bar: 20 μm.

In general, the mitochondrial import of proteins to the matrix depends on the presence of an N-terminal MTS of the target protein. The MTS recognizes the TOM receptor complex and transfers the protein as unfolded or partially unfolded polypeptide through the TOM complex and the TIM complex. Reaching the matrix, a cascade of metallopeptidases processes the imported polypeptide, starting with the mitochondrial processing peptidase MPP. Subsequently, mitochondrial chaperones support the refolding of the processed polypeptide to a functional protein. As a first step towards developing an optogenetic system to control the mitochondrial import of a protein of interest (POI), we compared the well-characterized MTS of COX8, a subunit of the respiratory chain complex IV [35], and the short artificial MTS, MTS_AMTS_, a variant of a cell-penetrating artificial-targeting peptide [36] (Figure 2a). MPP cleaves the first 23 residues of MTS_COX8_, comprising 28 residues, and further processing depends on the sequence fused to it. In the case of MTS_AMTS_, MPP releases the first 8 residues of the 11 residues of MTS_AMTS_. As proteins of interest, we used the fluorescent protein eGFP (239 amino residues) and the SNAP-tag protein (182 amino acid residues), a self-labeling variant of the human enzyme *O*^6^-alkylguanine DNA alkyltransferase used for the efficient labeling of fusion proteins with synthetic reporter molecules [37]. The expression of MTS_COX8_-eGFP in HEK 293T cells resulted in two protein bands with molecular weights according to an uncleaved MTS_COX8_-eGFP and a protein with a molecular weight slightly lower than eGFP (Figure 2b). However, MPP cleavage should result in a cleavage product slightly larger than eGFP because of 5 residues remaining from the MTS. This result hints at further processing steps following cleavage by MPP. In the case of the short MTS_AMTS_, a higher resolution obtained via appropriate gel electrophoresis conditions enabled the detection of a protein corresponding to the uncleaved MTS_AMTS_-eGFP and a cleavage product with the size of eGFP (Figure S1). Analyses of variants of the AMTS sequence revealed sequences with further-improved MPP cleavage. In the case of MTS_COX8_-SNAP, the processed protein was slightly larger than SNAP, which can be explained by the residues that remain fused to SNAP after the removal of MTS_COX8_ by MPP (Figure 2b). If an MTS mediates the import of a POI into mitochondria, the cleavage of the MTS by MPP should correlate with the mitochondrial localization of the POI.

Cell fractionation confirmed that the expression of MTS_COX8_-eGFP resulted in eGFP as a cleavage product, which localized with the mitochondrial fraction (Figure 2c). However, in the cytosolic fraction, uncleaved and cleaved products were detectable. This indicates an overload of the mitochondrial import system in this protein overexpression system. Clogging of the transport machinery with preproteins only partially imported into the matrix may release proteins back to the cytosol after MPP processing. The expression of MTS_AMTS_-eGFP provided no clear result, probably because the unprocessed and processed form could not be separated properly. Therefore, MTS_AMTS_ was not further included in this study. Similar to MTS_COX8_-eGFP, the expression of MTS_COX8_-SNAP showed processed SNAP in the mitochondrial fraction. The detection of the processed form in the cytosolic fraction indicates an overload of the mitochondrial import also in case of MTS_COX8_-SNAP. As expected, eGFP and SNAP, without MTS, were cytosolic proteins. In addition to cell fractionation, confocal microscopy studies were performed. And indeed, the expression of MTS_COX8_-SNAP, but not SNAP without MTS, resulted in a SNAP protein colocalizing with the mitochondrial network in living cells, with the MitoTracker used as a reporter for the visualization of the mitochondrial network (Figure 2d). Taken together, these results indicate that MTS_COX8_ is an appropriate sequence with which to target a POI to mitochondria and to allow for mitochondrial import as indicated by MPP processing.

### 3.2. Light-Induced Mitochondrial Import Based on the Two Plasmids System

**Figure 3:**
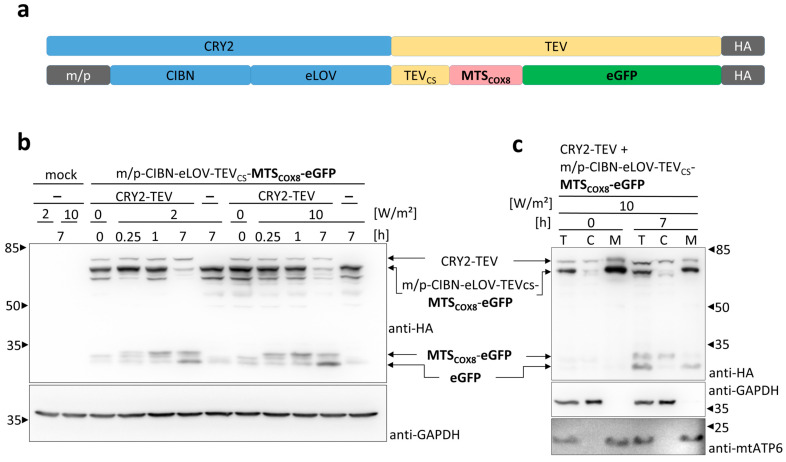
Light-induced mitochondrial protein import based on the two plasmids system: (**a**) Constructs used for the two-plasmids-based OptoMitoImport system. CRY2, cryptochrome 2; TEV, tobacco etch virus protease; m/p, myristoylation/palmitoylation signal corresponding to the N-terminal 12 residues of LYN [26]; CIBN, CRY2-interacting binder N-terminus; eLOV, improved light–oxygen–voltage-sensing domain; TEVcs, TEV cleavage site; MTS, mitochondrial targeting sequence; POI, protein of interest; HA, HA-tag. (**b**) HEK 293T cells were transfected with the constructs, as indicated, and lysed 27 h after transfection. Before lysis, the cells were exposed to 465 nm light (optoPlate) with an intensity of 2 W/m^2^ or 10 W/m^2^ for the time period as indicated or incubated in the dark. Samples were subjected to SDS-PAGE followed by immunoblotting with anti-HA antibody and anti-GAPDH antibody as the loading control. (**c**) After 23 h transfection, HEK 293T cells were illuminated for 4 h with 465 nm light (optoPlate; 10 W/m^2^) or incubated in the dark. Proceeding from total cell lysates (T), cytosolic (C) and mitochondrial (M) fractions were prepared. Samples were subjected to SDS-PAGE and immunoblotted with anti-HA antibody and anti-GAPDH as loading control or anti-GAPDH antibody and anti-mtATP6 as markers for the cytosolic and the mitochondrial fraction, respectively. Each point shown in the experiments is repeated at least three times, albeit in different sets of an experiment.

Next, we built up an optogenetic system for the control of the release of an MTS-POI fusion protein. (Figure 1). The system is composed of two components (Figure 3a): CRY2 fused to the TEV protease as well as the membrane-anchored fusion construct carrying CIBN for recruiting CRY2-TEV, eLOV for controlling the accessibility to TEVcs and MTS_COX8_-linked POI. The expression of both constructs should release MTS-POI on demand upon exposure to blue light and should subsequently enables its mitochondrial import. In a first set of experiments, we expressed both constructs in HEK 293T cells with eGFP as the POI (Figure 3b). Exposure of the cells to blue light 24 h after transfection resulted in TEV cleavage of the membrane-anchored fusion construct and the release of MTS_COX8_-eGFP within 15 min. Longer periods of illumination further decreased the amount of membrane-anchored protein, correlating with an increase in the TEV cleavage product MTS_COX8_-eGFP within 1 h. Upon 7 h of illumination, the membrane-bound fusion construct was almost completely cleaved by the TEV protease, and a protein the size of eGFP appeared, indicating the mitochondrial import and processing of the TEV cleaved MTS_COX8_-eGFP via MPP. The use of a higher light intensity, 10 W/m^2^, compared to 2 W/m^2^, accelerated the TEV cleavage process; however, it did not result in higher levels of MPP processed eGFP. Similar results were obtained using SNAP as the POI (Figure S2). The fractionation of light-exposed cells confirmed the presence of TEV-cleaved MTS_COX8_-eGFP in the cytosolic fraction and the further-processed eGFP in the mitochondrial fraction (Figure 3c). These data contradict the experiments showing an overloading of the mitochondrial machinery for the non-opto approach with MTS-POI. Thus, the two-plasmids-based OptoMitoImport enables the control of the mitochondrial import of the protein of interest very efficiently.

### 3.3. Light-Induced Mitochondrial Import Based on Bicistronic Constructs

**Figure 4:**
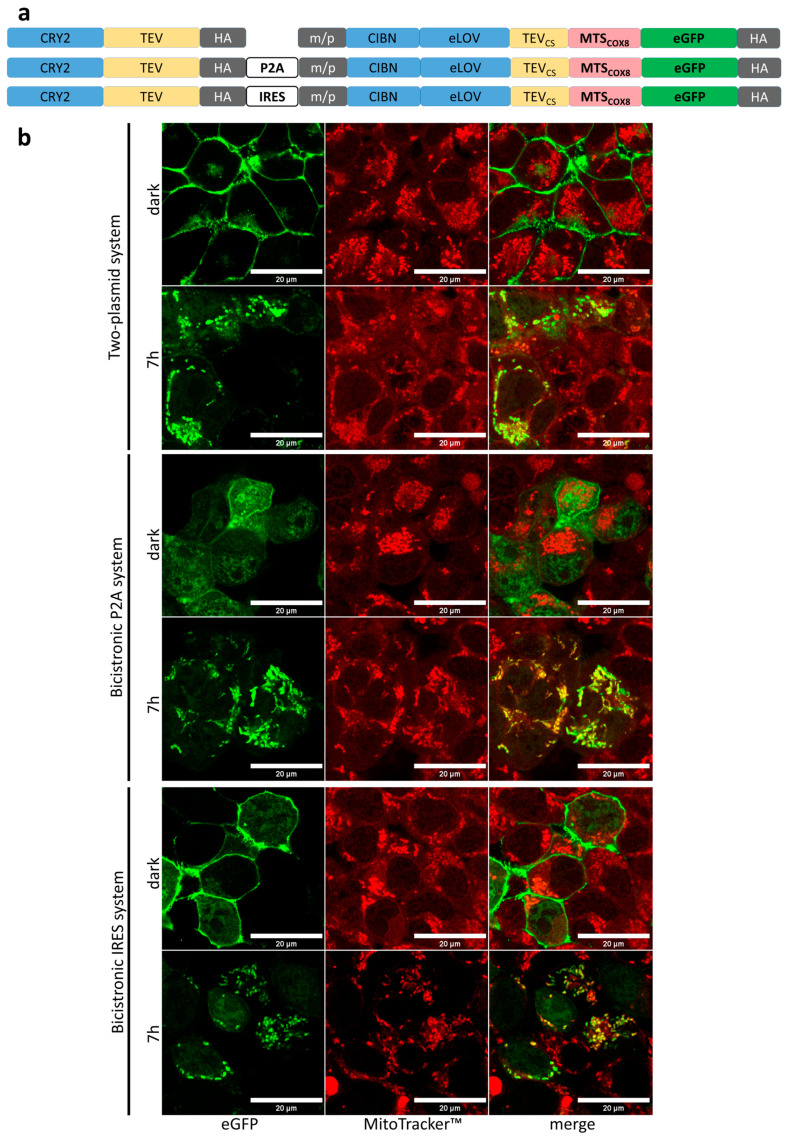
Light-induced release of eGFP from the plasma membrane and co-localization with mitochondria: (**a**) Constructs used for the OptoMitoImport systems. CRY2, cryptochrome 2; TEV, tobacco etch virus protease; P2A, 2A peptide sequence mediating ribosomal skipping [38]; IRES, internal ribosomal entry site [41]; m/p, plasma membrane targeting sequence [26]; CIBN, CRY2-interacting binder N-terminus; eLOV, improved light–oxygen–voltage-sensing domain; TEVcs, TEV cleavage site; MTS, mitochondrial targeting sequence; POI, protein of interest; HA, HA-tag. (**b**) HEK 293T cells transfected with the OptoMitoImport constructs were cultivated for 36 h before being exposed to 450 nm light for 7 h. Before fixation with paraformaldehyde and analysis via confocal fluorescence microscopy, the cells were incubated with MitoTracker TM Red CMXRos dye for the visualization of the mitochondrial network in red in living cells. Cultivation and imaging were performed in Ibidi µ-Slide 8 Well Grid-500. Co-localization is visualized by merging the images. Scale bar: 20 μm.

To simplify the light-controlled mitochondrial import system, we generated bicistronic systems in which the two components of OptoMitoImport are expressed from one plasmid, and the cistrons encoding these two components were separated by a P2A or an IRES sequence (Figure 4a). P2A, 19 amino acid residues in length, is a member of a class of viral oligonucleotides that induce ribosomal skipping during the translation of mRNAs [38], resulting in two polypeptides equally expressed: CRY2-TEV and the membrane-anchored component carrying MTS_COX8_-eGFP in our system. IRES, the internal ribosomal entry site, is a non-coding RNA element that allows for cap-independent translational initiation at the 5′ end of viral mRNA [39,40]. The insertion of an IRES element between two cistrons leads to the generation of a bicistronic complex and enables the translation of two cistrons from one transcript in eukaryotic cells [41]. First, we visualized the localization of eGFP depending on light exposure in the two-plasmid system, the bicistronic P2A system, and the bicistronic IRES system (Figure 4b). In all three systems, eGFP localized to the plasma membrane in the dark. Although there is some cytoplasmic eGFP detectable in the case of the P2A system, cytoplasmic eGFP did not colocalize with the mitochondria. Illumination of the cells for 7 h resulted in clear co-localization of eGFP with mitochondria, indicating the light-induced TEV cleavage of the membrane-anchored fusion construct and the release of eGFP equipped with an MTS. Illumination for 1 h only partially induced the release of eGFP from the plasma membrane and, accordingly, showed less co-localization with mitochondria (Figure S3). The data confirm the functionality of the OptoMitoImport tools concerning the light-induced release of eGFP from the plasma followed by co-localization with mitochondria.

**Figure 5:**
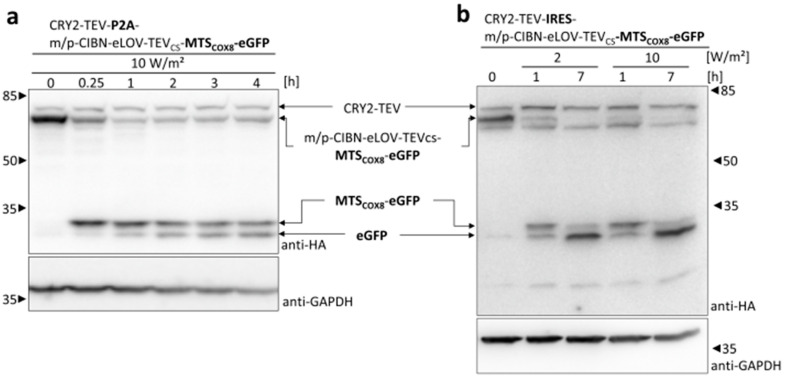
Light-induced mitochondrial protein import based on the bicistronic P2A and IRES systems: (**a**) HEK 293T cells were transfected with the bicistronic P2A construct and lysed 27 h after transfection. Before cell lysis, the cells were exposed to 465 nm light with an intensity of 10 W/m^2^ for the time period as indicated or incubated in the dark. Samples were subjected to SDS-PAGE followed by immunoblotting with anti-HA antibody and anti-GAPDH antibody as the loading control. (**b**) HEK 293T cells were transfected with the bicistronic IRES construct and lysed 27 h after transfection. Before cell lysis, the cells were exposed to 465 nm light with an intensity of 2 or 10 W/m^2^ for the time period as indicated or incubated in the dark. Samples were subjected to SDS-PAGE followed by immunoblotting with anti-HA antibody and anti-GAPDH antibody as the loading control. Each point shown in the experiments is repeated at least three times, albeit in different sets of an experiment.

To characterize the bicistronic systems in more detail, cells transfected with the bicistronic constructs were exposed to 465 nm light with an intensity of 10 W/m^2^ for different time periods (Figure 5). Upon exposure to blue light for 15 min, the level of the membrane-anchored construct decreased, and the TEV cleavage product MTS_COX8_-eGFP was clearly detectable in cells expressing the P2A construct (Figure 5a). Even a faint band of eGFP, the product of MPP processing in the mitochondrial matrix, could be detected. Prolonged illumination of the cells up to 4 h further elevated the level of eGFP, correlating with a decrease in the amount of MTS_COX8_-eGFP. Compared to 10 W/m^2^, exposure of the cells with a lower light intensity of 2 W/m^2^ decelerated the process of TEV cleavage. However, after 4 h of illumination, a similar amount of eGFP was produced (Figure S4). Similar results were obtained for the bicistronic IRES construct (Figure 5b). After 1 h of light exposure, the TEV cleavage product MTS_COX8_-eGFP was clearly detectable, as well as a faint band of MPP-processed eGFP. Illumination for 7 h led to almost complete loss of the membrane-anchored fusion protein and a strong band of MPP-processed eGFP.

The reversibility of the OptoMitoImport system depends on the light-controlled recruitment of the TEV to the membrane-anchored fusion construct. The precursor protein is synthesized continuously and can accumulate at the plasma membrane when cells are cultivated in the dark. Equally, the imported payload is continuously degraded depending on its half-life. To test for reversibility, cells expressing the bicistronic IRES construct were exposed to light followed by dark periods of different lengths to allow for the resynthesis of the precursor. Afterwards, the cells were illuminated again to enable light-induced TEV cleavage (Figure S5). Prolonged dark periods decreased the level of the TEV cleavage product MTS_COX8_-eGFP (Figure S5, lanes 4, 7 and 10). Accordingly, a second light period elevated the level of the TEV cleavage product MTS_COX8_-eGFP (Figure S5, lanes 5, 8 and 11). These data indicate the reversibility of the OptoMitoImport system mediated by the light-regulated TEV.

### 3.4. Light-Induced Mitochondrial Import of MTS Fusion Proteins Depends on the Membrane Potential across the Inner Mitochondrial Membrane

**Figure 6:**
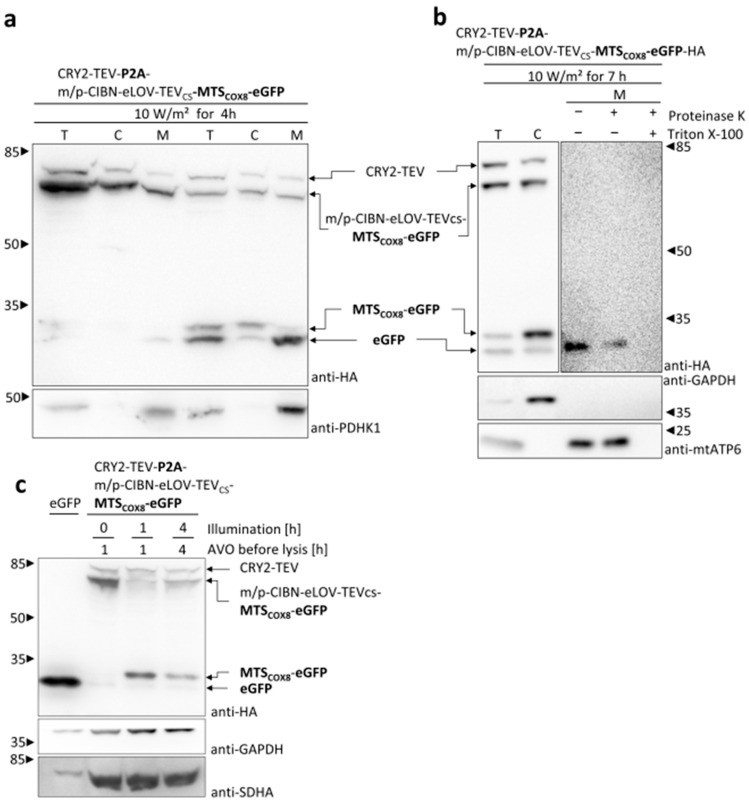
Characterization of the MTS-dependent mitochondrial import: (**a**) Twenty-three hours after being transfected, HEK 293T cells were illuminated for four hours with 465 nm light (10 W/m^2^) or incubated in the dark. Subsequently, total cell lysates (T), cytosolic (C) and mitochondrial (M) fractions were prepared by subcellular fractionation. Samples were subjected to SDS-PAGE and immunoblotted with anti-HA antibody, as well as anti-PDHK1 antibody as a marker for the mitochondrial fraction. (**b**) Twenty-four hours after transfection, HEK 293T cells were illuminated with 465 nm light (optoPlate) and 10 W/m^2^ for four hours. Subsequently, total cell lysates (T), cytosolic (C), and mitochondrial (M) fractions were prepared. The mitochondrial fraction was divided into three parts: one part remained untreated, while the second part and the third part were treated with 20 μg/mL proteinase K with or without 0.5% Triton X-100 for 15 min at RT, respectively. Cell lysates were subjected to SDS-PAGE and immunoblotted with anti-HA antibody, as well as anti-GAPDH antibody and anti-mtATP6 antibody as markers for the cytosolic and the mitochondrial fraction, respectively. (**c**) Light-induced mitochondrial import of MTS fusion proteins depends on the membrane potential across the inner mitochondrial membrane. HEK 293T cells were transfected with the bicistronic construct. Before lysis, cells were exposed to light of 450 nm with an intensity of 10 W/m^2^ for the time period indicated or incubated in the dark. The treatment with a mixture of 0.8 µM antimycin A, 0.05 µM valinomycin, and 2 µM oligomycin (AVO) was started shortly before exposure to 450 nm light (10 W/m^2^) for 1 or 4 h or, in case of the dark control, 1 h before cell lysis. Cell lysates were subjected to SDS-PAGE and immunoblotted with anti-HA, as well as anti-GAPDH antibody and anti-SDHA antibody as markers for the cytosolic and the mitochondrial fraction, respectively. The eGFP sample has been diluted to a fifth to prevent overloading.

For further characterization of the MTS-dependent mitochondrial import and to demonstrate the functionality of the OptoMitoImport tool, several approaches were performed. Cell fractionation experiments confirmed that the TEV cleavage product MTS_COX8_-eGFP was located in the cytosolic fraction and eGFP generated via MPP processing in the mitochondrial fraction (Figure 6a). These data proved the sequential processing of the membrane-anchored construct by the TEV upon blue light exposure and, subsequently, the processing of the cleavage product MTS_COX8_-eGFP to eGFP by matrix-localized MPP. To confirm the matrix localization of the imported protein, the mitochondrial fraction was treated with proteinase K. Proteins imported into the matrix should be protected from proteolytic digestion, whereas proteins only associated with mitochondria should be degraded.

Although not as efficient as the mitochondrial-encoded matrix protein mtATP6, MPP-processed eGFP is partially protect from proteinase K digestion. Co-treatment of the mitochondrial fraction with proteinase K and the detergent Triton X-100 resulted in the loss of eGFP and the matrix marker protein mtATP6 (Figure 6a,b). Taken together, these data confirm the consecutive cleavage via light-induced TEV cleavage and the MPP processing of proteins imported into the mitochondrial matrix. Thus, the bicistronic plasmid-based system worked convincingly and could be applied for kinetic experiments regarding the two-step process of OptoMitoImport.

The presequence pathway of the mitochondrial import strictly depends on the presence of the membrane potential across the inner mitochondrial membrane generated by the respiratory chain complexes. To verify whether the mitochondrial import by the OptoMitoImport system also depends on the membrane potential, the proton gradient was depleted by a mixture of the respiratory chain inhibitors antimycin, valinomycin, and oligomycin (AVO). Cells transfected with the bicistronic construct were treated with AVO at the time of beginning of the illumination, or, in the case of the dark sample, 1h prior to cell lysis (Figure 6c).

Depletion of the membrane potential by the respiratory chain inhibitors did not interfere with the cleavage by TEV. However, the TEV cleavage product MTS_COX8_-eGFP was not further processed to eGFP, indicating a lack of mitochondrial import correlating with the loss of MPP processing. These data demonstrate that OptoMitoImport is an appropriate tool with which to investigate the presequence pathway of a POI.

## 4. Discussion

In this study, we developed an optogenetic system to control the mitochondrial import of proteins mediated by the presequence pathway. OptoMitoImport is based on a two-step process: first, the release of MTS-POI via the cleavage of the TEV in a blue-light-dependent manner; and second, targeting the POI to the TOM receptor and importing into the mitochondrial matrix via MTS. Of the two presequences tested, MTS_COX8_ with 28 amino acid residues was more efficient and reliable than MTS_AMTS_ with 11 residues. One reason could be that longer presequences promote the matrix import more efficiently [42]; however, optimization of a specific MTS can also improve the mitochondrial import (see Figure S1). In addition to a two-plasmid-based system that carries CRY2-TEV on one plasmid and the membrane-anchored fusion construct comprising a light-dependent caged TEVcs and MTS-POI on the second, bicistronic constructs were generated in which both components were expressed from one plasmid and separated via a P2A or an IRES sequence. All three OptoMitoImport systems worked equally well concerning light-induced TEV cleavage and the processing of MTS_COX8_ by MPP. While the bicistronic systems enable the translation of fixed ratios of both components of OptoMitoImport [38,41], the two-plasmid-based system is more flexible concerning the titration of a specific MTS-POI. Importantly, the OptoMitoImport systems did not show any overloading effect of the mitochondrial machinery, in contrast to the overexpression of MTS-POI constructs.

The import of a protein into the mitochondrial matrix mediated by the presequence pathway depends on the membrane potential across the inner mitochondrial membrane [15]. Accordingly, depleting the membrane potential by AVO, inhibitors of the respiratory chain complexes disturbed the mitochondrial import, as indicated by the lack of MTS_COX8_-eGFP processed to eGFP by matrix peptidases. The first peptidase in the hierarchy of the peptidase cascade processing matrix-imported proteins is MPP. Further peptidases, such as XPNPEP3/Icp55 and MIP, may have trimmed the imported protein to a stable form that is subsequently refolded by matrix chaperones to a functional protein [18,43]. as indicated by the slightly reduced site of the processed eGFP (see Figure 2c). While the processing of imported MTS_COX8_ was clearly demonstrated by the appearance of eGFP, the role of sequential processing by different mitochondrial peptidases is not the topic of this study.

The mitochondrial import has been described as a co-translational as well as a post-translational process. In the case of a co-translational process, polypeptides synthesized at ribosomes associated with the outer mitochondrial membrane could directly contact the TOM receptor complex in an unfolded state, facilitating mitochondrial import. This process is well-documented for the dual targeting of proteins that are imported into the mitochondria but also reverse translocated to the cytosol [19]. However, the presequence pathway seems to be mainly a post-translational process. Accordingly, coincubation of the isolated mitochondria and the recombinant POI conjugated with an MTS leads to an enriched POI in the mitochondrial matrix in vitro, similar to recombinant proteins microinjected into the cytosol of mammalian cells [44]. Even a cell-membrane-penetrating artificial mitochondrial-targeting peptide enables the import of eGFP and metallothionein into mitochondria [36].

The OptoMitoImport system involves the light-induced cleavage of a membrane-targeted fusion protein by the TEV. At the time of light exposure, the TEV cleaves this precursor protein within 15 min, meaning that this step does not depend on newly synthesized proteins but is a post-translational step. The duration of light exposure and the light intensity applied control the efficiency of TEV cleavage. However, the mitochondrial import of the TEV cleavage product only depends on a functional MTS and the proper cellular conditions, such as an intact membrane potential at the inner mitochondrial membrane [45,46]. Therefore, the presence of MPP-processed eGFP demonstrates the mitochondrial import and the functionality of the system. The starting point and the efficiency of the system can be controlled by light. Regarding the light-controlled recruitment of the TEV to the precursor protein, the OptoMitoImport system is reversible. Incubation of the cells in darkness result in enrichment of membrane-anchored precursor protein and degradation of mitochondrial proteins, depending on their half-life.

Recently, Berry and Wojtovich engineered the system mt^SWTICH^ based on genetically encoded, light-activated cation channels or proton pumps [47]. mt^SWTICH^ constructs modulate the PMF and thereby the energy availability in response to light and are tools with which to control mitochondrial function and cellular metabolism. While the mt^SWTICH^ targets the membrane potential to control mitochondrial functions in general, the OptoMitoImport uses an MTS for the import of a protein of interest via the presequence pathway.

## 5. Conclusions

OptoMitoImport offers a generic approach to study the presequence pathway of the mitochondrial import. OptoMitoImport can be applied to study the post-translational mitochondrial protein import as it clearly depends on a preformed membrane-attached precursor fusion construct. The system uses light as an inducer and works independently of external triggers. Studies of the mitochondrial phosphoproteome linked mitochondrial AKT with hypoxic tumor reprogramming [48]. Thus, OptoMitoImport may be used as a tool with which to study the function of mitochondrial AKT in tumor development [49,50].

## Data Availability

The data presented in this study are available upon request from the corresponding authors.

## Supplementary Materials

The following supporting information can be downloaded at https://www.mdpi.com/article/10.3390/cells13191671/s1. Figure S1 (related to Figure 2). Analysis of AMTS variants. Figure S2 (related to Figure 3). Light-induced mitochondrial protein import of MTS_AMTS_-SNAP. Figure S3 (related to Figure 4). Light-induced release of eGFP from the plasma membrane and co-localization with mitochondria. Figure S4 (related to Figure 5). Influence of the light intensity on TEV cleavage and the mitochondrial protein import. Figure S5 (related to Figure 5). Reversibility of the OptoMitoImport system. Table S1. Plasmids used in this study.
