# Supplementary material for: Optogenetic Control of the Mitochondrial Protein Import in Mammalian Cells
Source: Cells. 2024 Oct 9;13(19):1671. doi: 10.3390/cells13191671 (PMC11482626; doi:10.3390/cells13191671)
Supplement: Supplementary file 1 [file cells-13-01671-s001.zip › cells-3203701-supplementary.pdf]

Supplementary Materials

Optogenetic Control of the Mitochondrial Protein Import in Mammalian cells

Lukas F. J. Althoff [1,†], Markus M. Kramer [1,2,†], Benjamin Bührer [1,2,‡], Denise Gaspar [1] and Gerald Radziwill [1,*]

[1] Faculty of Biology and Signalling Research Centres BIOSS and CIBSS, University of Freiburg, 79104 Freiburg, Germany; lukas.althoff@biologie.uni-freiburg.de (L.F.J.A.); markus.kramer@cibss.uni-freiburg.de (M.M.K.); benjamin.buehrer@uniklinik-freiburg.de (B.B.), denise.gaspar@bioss.uni-freiburg.de (D.G.); gerald.radziwill@biologie.uni-freiburg.de (G.R.)

[2] Spemann Graduate School of Biology and Medicine (SGBM), University of Freiburg, 79104 Freiburg, Germany

* Correspondence: gerald.radziwill@biologie.uni-freiburg.de; Tel.: +49-761-203-2635

[†] These authors contributed equally to this work

[‡] Current address: Department of Internal Medicine I, Faculty of Medicine, University of Freiburg, 79106 Freiburg, Germany

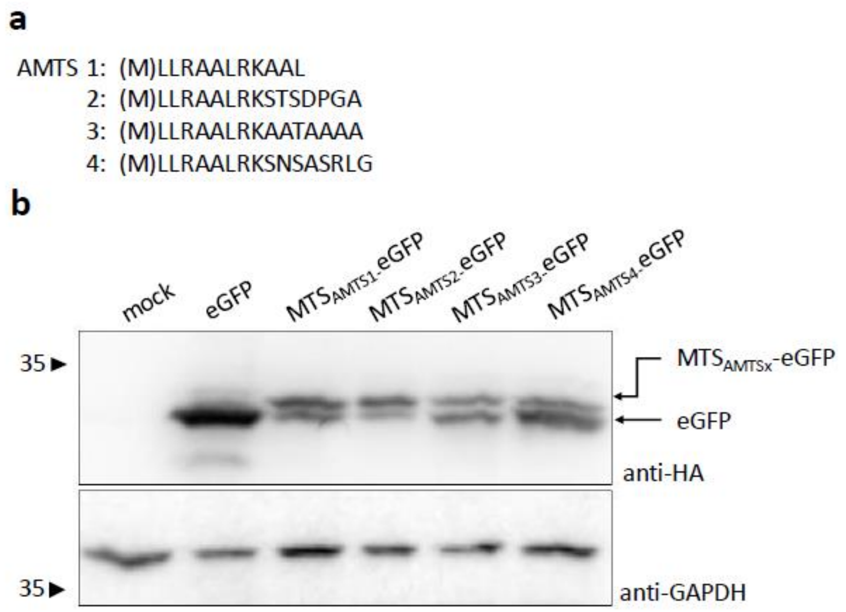

Figure S1 (related to Fig.2). Analysis of AMTS variants: (a) Sequence comparison of the AMTS variants tested in this study; M; methionine at the translational start site. (b) HEK 293T cells were transfected with the constructs as indicated. 24 h after transfection, the cells were lysed and the samples were subjected to SDS-PAGE followed by immunoblotting with anti-HA antibody and anti-GAPDH antibody as loading control. For higher resolution of the samples a 12% SDS-PAGE was performed.

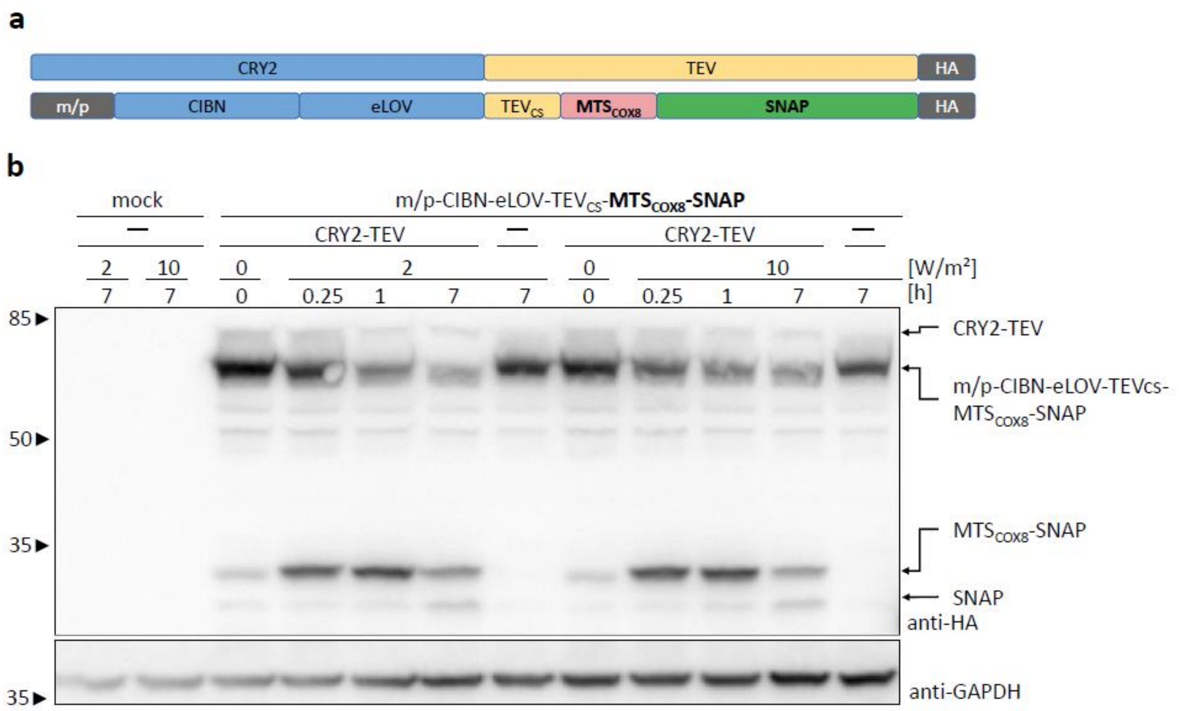

Figure S2 (related to Fig. 3). Light-induced mitochondrial protein import of MTS_{AMTS}-SNAP: (**a**) Constructs used for the two plasmids-based OptoMitoImport system with SNAP as protein of interest. CRY2, cryptochrome 2; TEV, tobacco etch virus protease; m/p, myristoylation/palmitoylation signal corresponding to the N-terminal 12 residues of LYN; CIBN, CRY2-interacting binder N-terminus; eLOV, improved light-oxygen-voltage-sensing domain; TEVcs, TEV cleavage site; MTS, mitochondrial targeting sequence; SNAP, SNAP-tag (**b**) HEK 293T cells were transfected with the constructs as indicated and lysed 27 h after transfection. Before cell lysis, the cells were exposed to 465 nm light (optoPlate) with an intensity of 2 W/m^2 or 10 W/m^2 for the time period indicated or incubated in the dark. Samples were subjected to SDS-PAGE followed by immunoblotting with anti-HA antibody and anti-GAPDH antibody as loading control.

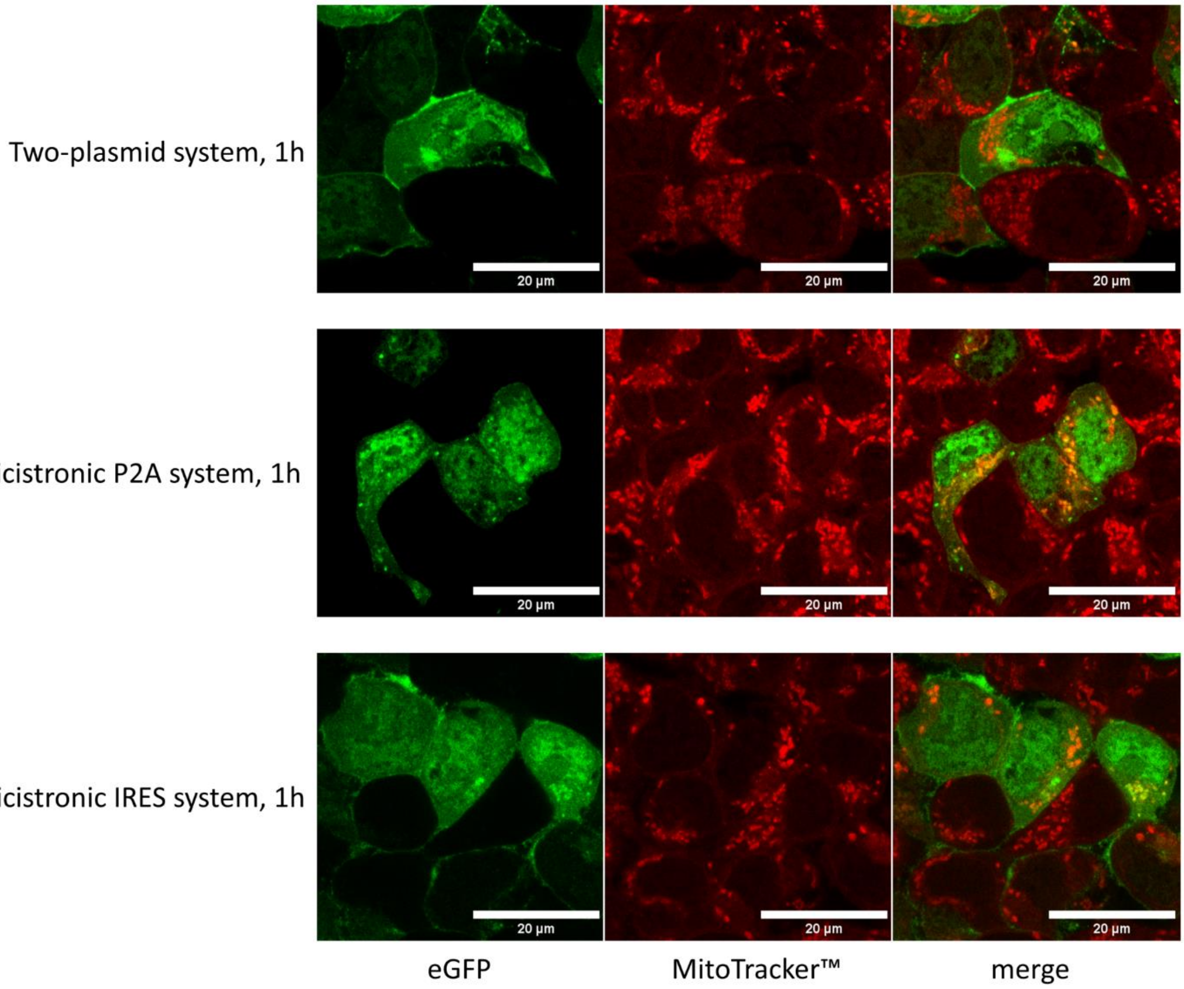

Figure S3 (related to Fig. 4). Light-induced release of eGFP from the plasma membrane and co-localization with mitochondria: HEK 293T cells transfected with the OptoMitoImport constructs were cultivated for 36 h, before exposed to 450 nm light for 1 h. Before fixation with paraformaldehyde and analysis by confocal fluorescence microscopy, the cells were incubated with MitoTracker TM Red CMXRos dye for the visualization of the mitochondrial network in red in living cells. Cultivation and imaging were performed in Ibidi µ-Slide 8 Well Grid-500. Co-localization is visualized by merging the images. Scale bar, 20 µm.

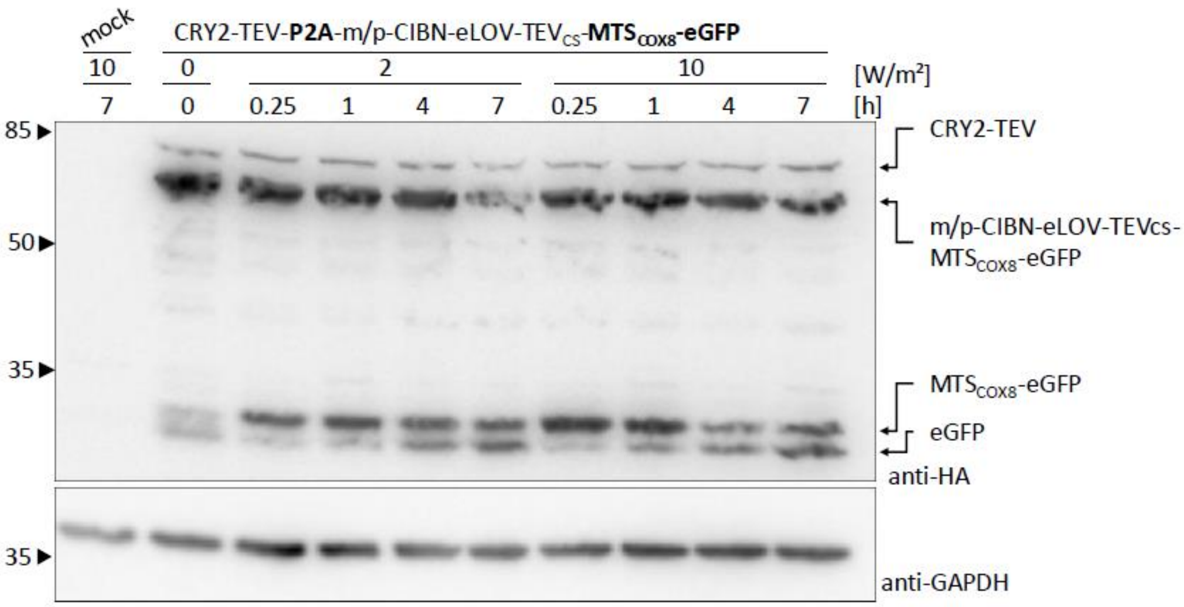

Figure S4 (related to Fig.5). Influence of the light intensity on TEV cleavage and the mitochondrial protein import: HEK 293T cells were transfected with the bicistronic P2A construct and lysed 27 h after transfection. Before cell lysis, the cells were exposed to 465 nm (optoPlate) light with an intensity of 2 W/m^2 or 10 W/m^2 for the time period as indicated or incubated in the dark. Samples were subjected to SDS-PAGE followed by immunoblotting with anti-HA antibody and anti-GAPDH antibody as loading control.

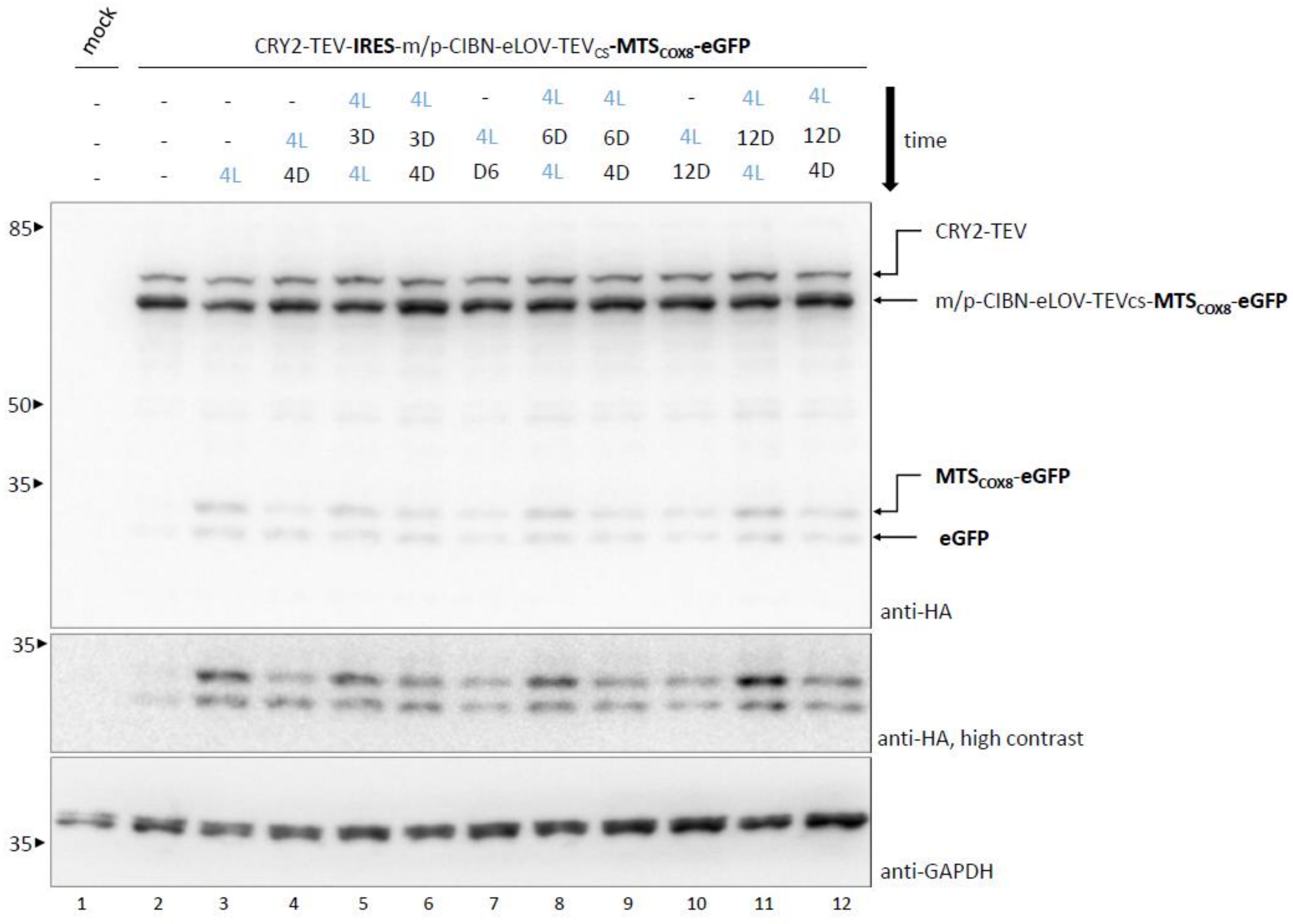

Figure S5 (related to Fig.5). Reversibility of the OptoMitoImport system: HEK 293T cells were transfected with the bicistronic IRES construct. The cells were exposed to different illumination scheme as indicated. and lysed 27 h after transfection. Before cell lysis, the cells were exposed to 465 nm (optoPlate) light with an intensity of 2 W/m^2 or 10 W/m^2 for the time period as indicated or incubated in the dark. Samples were subjected to SDS-PAGE followed by immunoblotting with anti-HA antibody and anti-GAPDH antibody as loading control.

Table 1: Plasmids used in this study

Name	construct
pMMK214	eGFP-HA
pMMK225	MTS_{COX8}-eGFP-HA
pMMK255	MTS_{AMTS}-eGFP-HA
pMMK562	MTS_{AMTS2}-eGFP-HA
pMMK564	MTS_{AMTS3}-eGFP-HA
pMMK566	MTS_{AMTS4}-eGFP-HA
pMMK557	SNAP-HA
pMMK558	MTS_{COX8}-SNAP-HA
pMMK172	CRY2-uTEVdelta-HA
pMMK228	m/p-CIBN-eLOV-TEVcs QMGGG-COX8-eGFP-HA
pMMK552	m/p-CIBN-eLOV-TEVcs-QMGGG-COX8-SNAP-HA
pMMK522	Cry2-TEV-HA-IRES(att)-m/p-CIBN-eLOV-COX8-eGFP-HA
pMMK556	CRY2-uTEV1delta-HA-P2A-m/p-CIBN-eLOV-TEVcs QMGGG-COX8-eGFP-HA
